# Effects of Gibberellic Acid and N, N-Dimethyl Piperidinium Chloride on the Dose of and Physiological Responses to Prometryn in Black Nightshade (*Solanum nigrum* L.)

**DOI:** 10.1371/journal.pone.0093654

**Published:** 2014-04-07

**Authors:** Hailan Jiang, Xiaoxia Deng, Jungang Wang, Jing Wang, Jun Peng, Tingting Zhou

**Affiliations:** 1 College of Agriculture, Shihezi University, Shihezi, Xinjiang, People’s Republic of China; 2 Kuerle Agricultural Technology Extension Center, Kuerle, Xinjiang, People’s Republic of China; University of Vigo, Spain

## Abstract

The use of gibberellic acid (GA_3_) and N, N-dimethyl piperidinium chloride (DPC) in combination with prometryn would likely increase the control of black nightshade in cotton fields. Experiments were designed to investigate the physiological and biochemical responses of black nightshade at the three- to four-leaf stage to prometryn applied at different rates, either alone or in combination with GA_3_ or DPC, in a greenhouse environment. These studies demonstrated that prometryn applied in combination with DPC at low rates (7.2 g ai ha^−1^) led to increased fresh weight and visible injury of black nightshade compared with prometryn applied alone or in combination with GA_3_; however, at rates of 36, 180, and 900 g ai ha^−1^, prometryn in combination with DPC caused the least visible injury among all treatments and prometryn in combination with GA_3_ caused the greatest visible injury. These results suggest that black nightshade suffered more severe damage when prometryn was applied in combination with GA_3_, which is supported by the reduced soluble protein content, lower antioxidant enzyme activities, and higher malondialdehyde (MDA) content in the plants treated with prometryn plus GA_3_. These results indicate that the application of GA_3_ in combination with prometryn to black nightshade may have the potential to lower the levels of prometryn tolerance in these plants.

## Introduction

Black nightshade (*Solanum nigrum* L.) is widely distributed throughout the world and is an important weed in many countries [1]. In recent decades, black nightshade has become increasingly problematic as a field crop weed [2], [3]. It is also one of the dominant weeds in cotton fields [4], [5] and seriously affects the growth of cotton.

Prometryn is a triazine herbicide in the same family as simetryn. Cotton is tolerant to certain triazine herbicides, such as prometryn, when they are applied at pre-emergence or post-emergence; however, severe injury can occur following post-emergence application [6], [7]. The triazine herbicides control of a wide spectrum of weeds in crop and non-crop areas under many environmental and edaphic conditions. Prometryn has been widely used as a residual soil-applied and post-emergence-directed herbicide in cotton [8], [9] and provides season-long control of annual grasses [10], [11].

Plant growth regulators, such as N, N-dimethyl piperidinium chloride (DPC) and gibberellic acid (GA_3_), are widely used in cotton production and have become indispensable tools in cotton cultivation [12], [13]. Although variations in the application of these growth regulators can occur among regions, years, and environmental conditions, they are applied throughout the growing season up to several weeks prior to harvest. DPC has been known to reduce the synthesis of GA_3_, resulting in the suppression of cell enlargement [14]. Thus, DPC-treated plants tend to be short and narrow with thick, small leaves [15], while GA_3_-treated plants present an opposite phenotype. Recently, there has been increased interest in the co-application of herbicides with plant growth regulators [16]. Applying these products in combination is also preferred by growers due to the convenience, reduced time investment, and reduced application and labour costs.

Due to the availability of prometryn-tolerant cotton, it is likely that this herbicide will be applied simultaneously with plant growth regulators and will effectively control black nightshade when it is applied to cotton at early post-emergence or late post-emergence [17], [18], [19]. Despite these benefits, incompatibility can occur with certain pesticides. Therefore, defining the interactions of agrochemicals is important when considering the simultaneous application of multiple agrochemicals [20], [21]. Because previous studies have seldom focused on the physiological responses of plants to the combined application of herbicides and plant growth regulators, it is desirable to devise an index of resistance that reflects physiological responses under different treatments. We hypothesised that the foliar application of plant growth regulators to black nightshade would strengthen the effect of prometryn on weed control. Therefore, the current study was designed to evaluate the efficacy of prometryn when applied alone or in combination with GA_3_ or DPC in controlling black nightshade and to study the physiological and biochemical response of the weed to co-application in a greenhouse.

## Materials and Methods

### Experimental Conditions and Treatments

The experiments were conducted in a greenhouse at Shihezi University in Xinjiang in 2012. The greenhouse temperature was maintained between 22°C at night and 29°C during the day. Separate experiments were conducted to investigate the control of black nightshade and to assess the physiological and biochemical changes in this plant. The plants were watered daily, grown under natural sunlight, and fertilised every 7 d to maintain active growth. Treatments for the black nightshade included 5 levels of the non-selective herbicide prometryn (7.2, 36, 180, 900, and 4500 g ai ha^−1^) applied alone or in combination with a plant growth regulator, either DPC (22.05 kg ai ha^−1^) or GA_3_ (14.7 kg ai ha^−1^). For the physiological and biochemical response study, the treatments consisted of prometryn (900 g ai ha^−1^) applied alone or in combination with a plant growth regulator, either DPC (22.05 kg ai ha^−1^) or GA_3_ (14.7 kg ai ha^−1^). All chemicals were applied at the manufacturer’s recommended rate. A non-treated control was included in these experiments. The experimental design for the control of black nightshade experiment and for the physiological and biochemical response experiment was a randomised complete block with four treatment replications.

Black nightshade seed samples (provided by Shihezi University) were stored in a cold room at 4°C prior to the initiation of the study. The black nightshade seeds were planted in a 20×15×5 cm plastic crisper in the greenhouse to accelerate germination. Once the black nightshade seedlings had germinated, 5 seedlings were transplanted into plastic pots (16 cm diameter, 11 cm height) filled with potting soil (fine-loamy, mixed, Typic Argiudoll) at pH 6.4 containing 1.9% organic matter. Three seedlings were removed prior to the treatments, and the treatments were applied when the black nightshade seedlings reached the three- to four-leaf stage.

### Dose Response Study

The black nightshade plants were excised at the soil level 6 d after the treatments to determine the fresh weight and calculate the percent reduction. Visual estimates of percent injury were recorded 6 d after treatment using a scale of 0 to 100, where 0 = no foliar injury and 100 = plant death. Foliar chlorosis, necrosis, tissue distortion, swelling, malformation, and plant stunting were considered when visually estimating the percent injury.

### Physiological and Biochemical Response Study

All the measurements were recorded close to midday on the uppermost fully expanded main stem leaf located three nodes below the terminus of the plant. The third main stem leaf was then collected 3 d and 6 d after application to observe changes in black nightshade physiological parameters, especially chlorophyll content, malondialdehyde content, and soluble protein content, as well as changes in biochemical parameters and antioxidant enzyme activity (superoxide dismutase (SOD), catalase (CAT), and peroxidise (POD)). Prior to the physiological and biochemical measurements, a single leaf of each plant was collected and immediately stored in an ultra freezer (−80°C) for antioxidant enzyme and protein extraction. A UV spectrophotometer (UV-1800; Shimadzu Co., Ltd., Japan) was used for measuring the changes in the physiological and biochemical parameters.

The chlorophyll (CHL) content of samples was measured using the method specified by Arnon [22]. Lipid peroxidation was defined as the content of all 2-thiobarbituric acid-reactive substances and was expressed as equivalents of MDA, as reported by Heath and Packer [23].

Frozen leaves (0.5 g) were homogenised in 50 mM potassium phosphate buffer (pH 7.8) containing 1 mM EDTA, 3 mM 2-mercaptoethanol, and 5% (w/v) polyvinylpyrrolidone (PVP) in a chilled mortar and pestle. The homogenate was centrifuged at 12,000×g for 20 min at 4°C, and the supernatant was used for enzyme assays.

Soluble proteins were measured according to the technique described by Bradford [24].

The SOD activity was assayed by the nitroblue tetrazolium (NBT) method [25]. The 3 ml reaction mixture contained 50 mM Na-phosphate buffer (pH 7.3), 13 mM methionine, 75 mM NBT, 0.1 mM EDTA, 4 mM riboflavin, and 0.2 ml of enzyme extract. This reaction was initiated by the addition of riboflavin, and the glass test tubes were shaken and placed under fluorescent lamps (160 μmol m^−2 ^s^−1^). The reaction was allowed to proceed for 5 min and was then stopped by switching off the light. The absorbance was measured at 560 nm. Blanks and controls were treated in the same manner but lacked illumination and enzyme, respectively.

The activities of POD and CAT were assayed using the method of Chance and Maehly [26] with some modifications. The POD reaction solution (3 ml) contained 50 mM phosphate buffer (pH 7.8), 25 mM guaiacol, 200 mM H_2_O_2_, and 0.5 ml of enzyme extract. Changes in the absorbance of the reaction solution at 470 nm were determined every 30 s. One unit of POD activity was defined as an absorbance change of 0.01 unit min^−1^. The CAT reaction solution (3 ml) contained 50 mM phosphate buffer (pH 7.0), 200 mM H_2_O_2_, and 0.5 ml of enzyme extract. The reaction was initiated by the addition of the extract. Changes in the absorbance of the reaction solution at 240 nm were measured every 30 s.

### Data Analysis

Differences between the plant growth regulator treatments were detected using analysis of variance (ANOVA). All data analyses were conducted using SPSS Statistics 17.0 with estimates of means and standard errors, and Duncan’s multiple range test was used for determining significant differences.

## Results

### Dose-response Study

**Figure 1:**
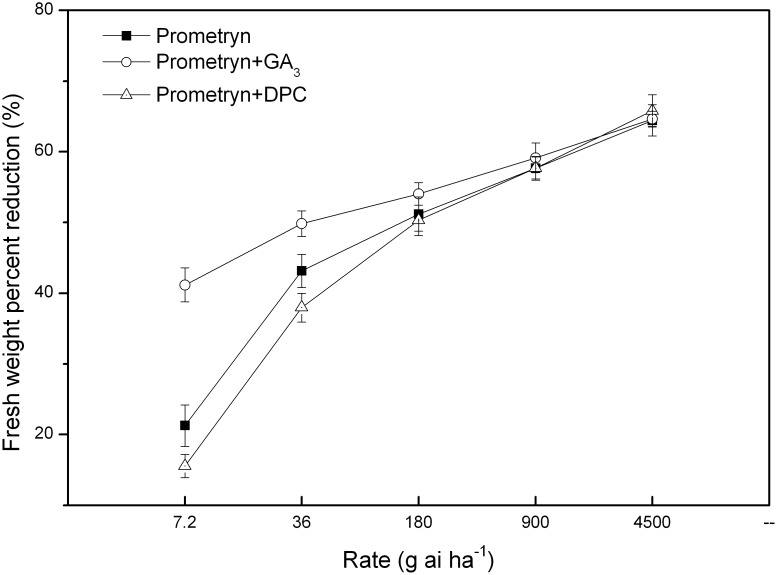
Percent reduction in fresh weight caused by prometryn applied alone or in combination with GA_3_ or DPC. Measurements were performed 6 d after application. The data are the means of 4 replicates ± standard error.

In general, the fresh weight observed at 6 d after application decreased as the herbicide rate increased, and the fresh weight was decreased to a similar extent across rates for all three treatments (Fig. 1). All herbicide treatments at rates of 7.2 and 36 g ai ha^−1^ caused reductions in fresh weight of less than 50%. The fresh weight reductions caused by prometryn and prometryn plus DPC were not drastically different. Prometryn applied in combination with GA_3_ at 7.2, 36, and 180 g ai ha^−1^ provided greater control than prometryn applied alone or combined with DPC; however, minimal differences were found when the rates of all treatments were higher than 180 g ai ha^−1^.

**Figure 2:**
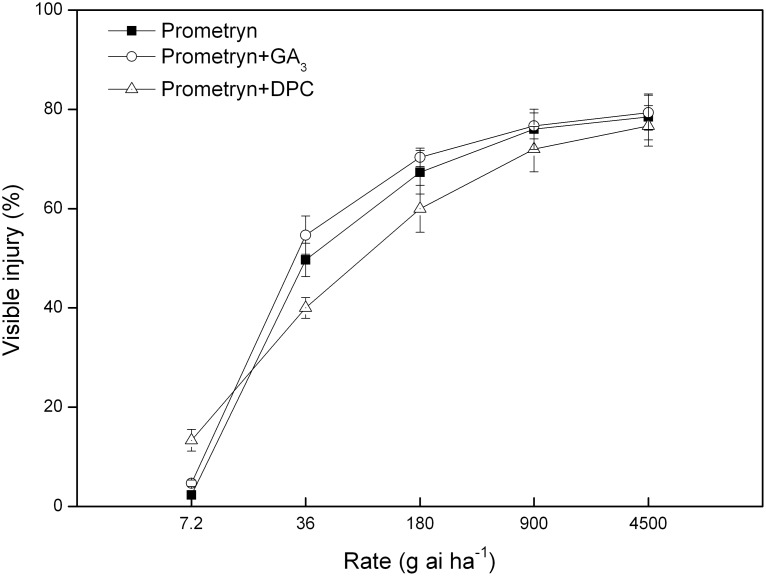
Visible injury caused by prometryn applied alone or in combination with GA_3_ or DPC. Measurements were performed 6 d after application. The data are the means of 4 replicates ± standard error.

Visible injury increased as the herbicide rates increased, with prometryn plus GA_3_ causing the greatest injury at 6 d after treatment (Fig. 2). All treatments caused less than 20% visible injury at 7.2 g ai ha^−1^. Prometryn combined with DPC at 7.2 g ai ha^−1^ provided the best control of black nightshade; however, it caused the least injury at 36 and 180 g ai ha^−1^. Although the visible injury caused by prometryn plus GA_3_ was more serious than the injury caused by prometryn alone, the visible injuries were similar at all rates.

### Physiological and Biochemical Response Study

**Figure 3:**
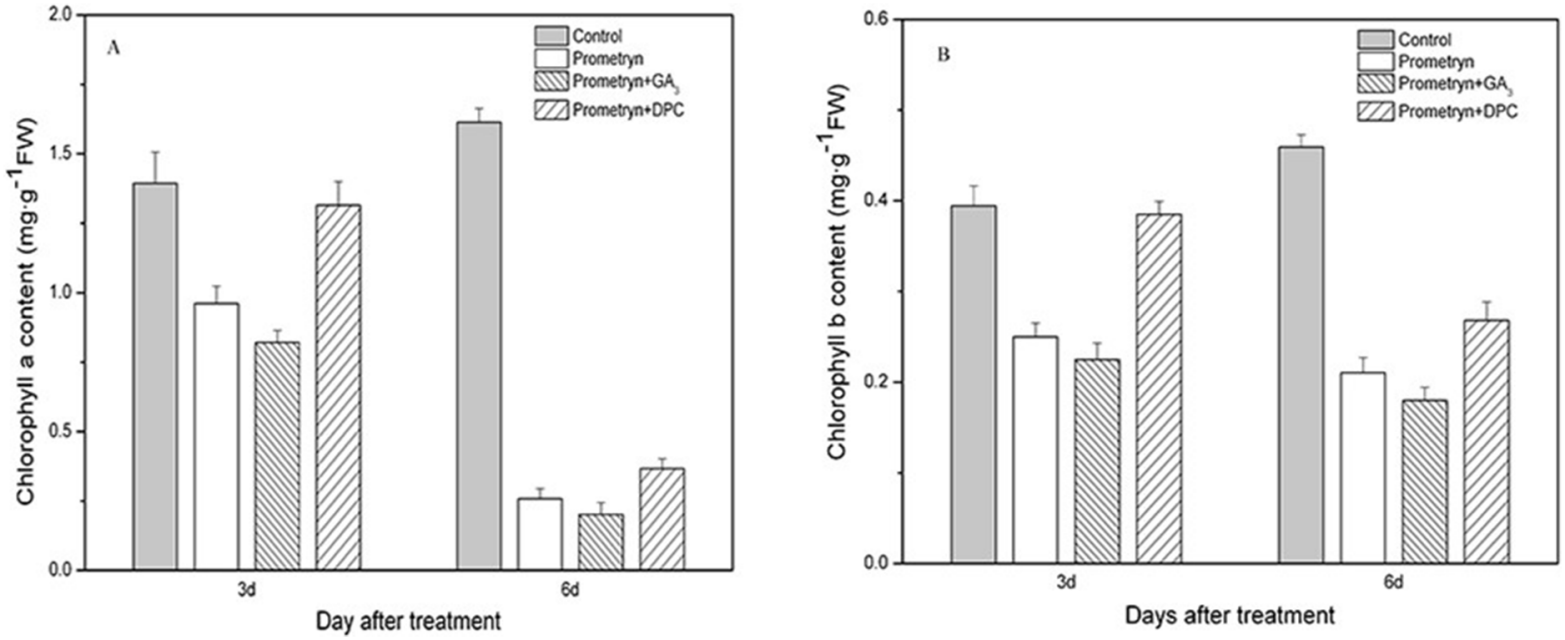
Effect of prometryn applied alone or in combination with GA_3_ or DPC on chlorophyll content. A: chlorophyll a content. B: chlorophyll b content. Measurements were performed 3 and 6 d after application. The data are the means of 4 replicates + standard error.

A similar trend was observed in the changes in chlorophyll a and chlorophyll b contents after all treatments. After the prometryn treatment, the chlorophyll levels decreased continuously and drastically by 31.09% (CHL a) and 36.61% (CHL b) 3 d after application and by 84.01% (CHL a) and 42.61% (CHL b) 6 d after application (Fig. 3). When prometryn was applied in combination with GA_3_, the change in chlorophyll content was more rapid, decreasing by 14.61% (CHL a) and 10.13% CHL b) 3 d after application and by 21.83% (CHL a) and 14.58% (CHL b) 6 d after application, compared to the treatment with prometryn alone (Fig. 3); however, 6 d after application, the effects of these two treatments were not significant. Although the chlorophyll content of the plants treated with prometryn plus DPC also decreased, the change was not significant compared to the control 3 d after application. The chlorophyll content of the plants treated with prometryn plus DPC 6 d after application was significantly lower than that of the control; however, it was still significantly higher than the chlorophyll content of plants treated with prometryn or prometryn plus GA_3_.

**Figure 4:**
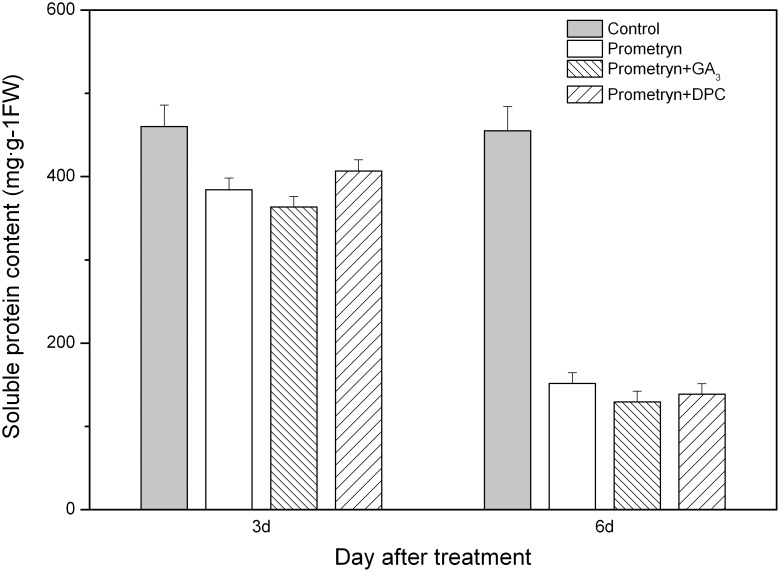
Effect of prometryn applied alone or in combination with GA_3_ or DPC on soluble protein content. Measurements were performed 3 and 6 d after application. The data are the means of 4 replicates + standard error.

The soluble protein content was significantly different between the prometryn and the control treatments 3 and 6 d after application (Fig. 4), and the soluble protein content of all treated plants was significantly lower than that of the untreated plants. The soluble protein content of plants treated with prometryn plus GA_3_ was significantly lower than that of plants treated with prometryn plus DPC 3 d after application; however, the soluble protein content of plants exposed to these treatments was not significantly different from that of plants treated with prometryn alone. No significant difference was observed in the soluble protein content of all prometryn-treated plants 6 d after application. The percent reductions in soluble protein content were 66.68%, 71.57%, and 69.49% for the prometryn, prometryn plus GA_3_, and prometryn plus DPC treatments, respectively, compared with the control.

**Figure 5:**
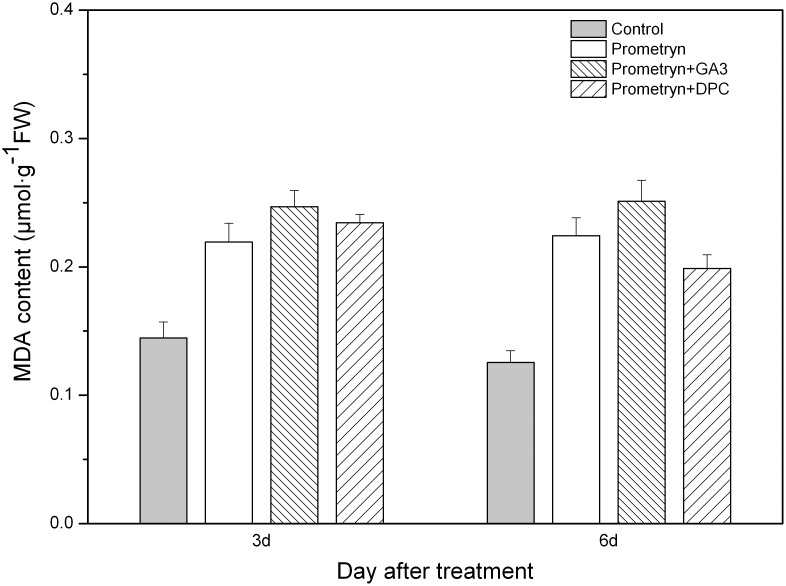
Effect of prometryn applied alone or in combination with GA_3_ or DPC on MDA content. Measurements were performed 3 and 6 d after application. The data are the means of 4 replicates + standard error.

After prometryn treatment, the MDA content of the plants increased drastically by 34.07% and 42.61% 3 d and 6 d after application, respectively (Fig. 5). When prometryn was applied in combination with GA_3_, the increase in MDA content was quicker than prometryn treatment alone. The highest MDA content was detected in plants treated with prometryn plus GA_3_ 3 d and 6 d after application; however, no significant differences were found between the prometryn and prometryn plus GA_3_ treatments. Compared with the control, the MDA content in plants treated with prometryn, prometryn plus GA_3_, and prometryn plus DPC was increased by 78.71%, 92.03%, and 48.82%, respectively.

### Changes in Antioxidant Enzyme Activities

**Figure 6:**
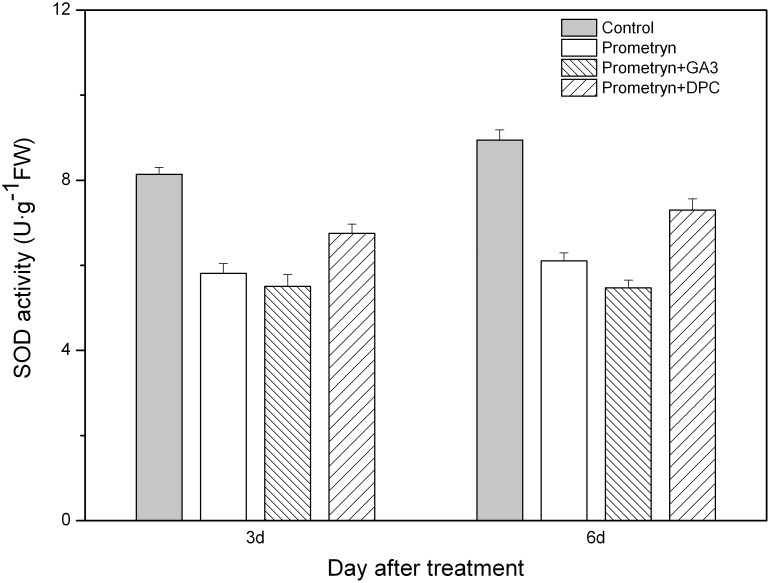
Effect of prometryn applied alone or in combination with GA_3_ or DPC on SOD activity. Measurements were performed 3 and 6 d after application. The data are the means of 4 replicates + standard error.

Prometryn applied alone or in combination with either GA_3_ or DPC had a significant effect on SOD activity; i.e., herbicide-treated plants exhibited significantly lower SOD activities compared with the control plants 3 d and 6 d after application (Fig. 6). The SOD activity of plants treated with prometryn plus DPC decreased 17.07% (3 d) and 18.37% (6 d) compared to the control. The lowest SOD activity was found in the plants treated with prometryn plus GA_3_; however, no significant difference was found between plants treated with prometryn alone and those treated with prometryn plus GA_3_.

**Figure 7:**
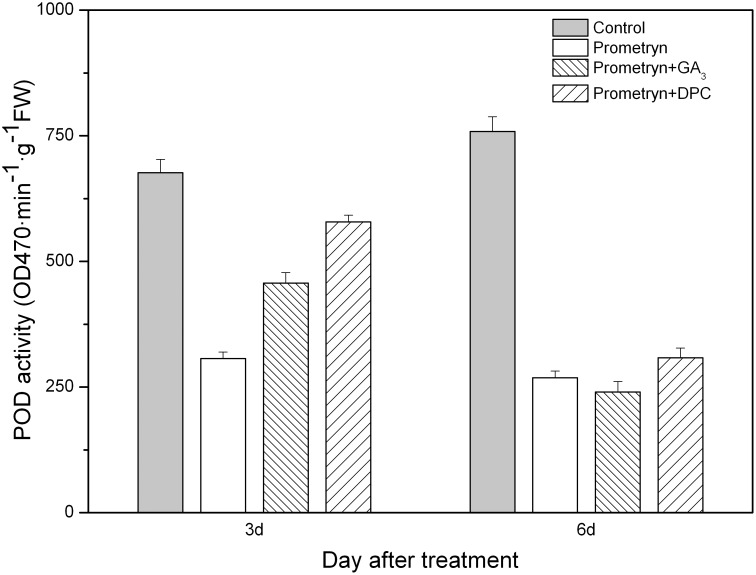
Effect of prometryn applied alone or in combination with GA_3_ or DPC on POD activity. Measurements were performed 3 and 6 d after application. The data are the means of 4 replicates + standard error.

Prometryn applied alone or in combination with either GA_3_ or DPC had a significant effect on POD activity; i.e., herbicide-treated plants exhibited significantly lower POD activities compared with the control plants 3 d and 6 d after application. However, no significant differences were observed between any of the herbicide treatments 6 d after application (Fig. 7). The POD activity of plants treated with prometryn plus DPC decreased 14.53% (3 d) and 59.34% (6 d) compared to the control. The lowest POD activities were noted in the plants treated with prometryn (3 d) and prometryn plus GA_3_ (6 d); in these plants, POD activity decreased by 54.68% and 68.35%, respectively, compared with the control.

**Figure 8:**
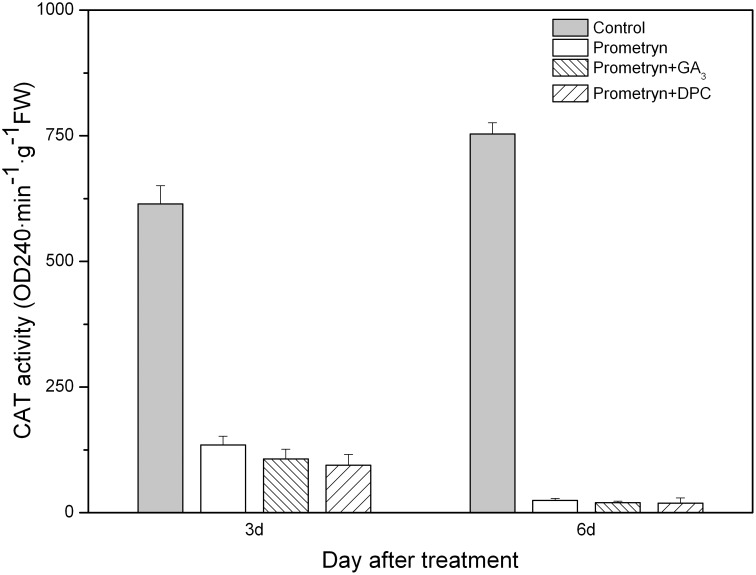
Effect of prometryn applied alone or in combination with GA_3_ or DPC on CAT activity. Measurements were performed 3 and 6 d after application. The data are the means of 4 replicates + standard error.

Prometryn applied alone or in combination with either GA_3_ or DPC also had a significant effect on CAT activity; i.e., the herbicide-treated plants exhibited significantly lower CAT activities compared with the control plants 3 d and 6 d after application. However, no significant difference was observed between any of the herbicide treatments 3 d and 6 d after application (Fig. 8). Compared to the control, the CAT activities in plants subjected to all treatments were decreased by more than 95%.

## Discussion

The proper use of growth-regulating chemicals requires a detailed understanding of the effects of their interaction with herbicides on plants. The results of this study suggest that the application of prometryn in combination with GA_3_ led to a greater reduction of plant fresh weight compared with prometryn alone and caused the greatest visual injury, indicating that prometryn had a stronger effect when it was combined with GA_3_. Changes in physiological and biochemical parameters after the foliar application of prometryn plus plant growth regulators help to explain the greater visible injury caused by prometryn in combination with GA_3_ in black nightshade.

CHL is vital for photosynthesis, which allows plants to obtain energy from light. The destruction and degradation of CHL directly results in a reduced photosynthetic rate and leads to leaf senescence. It has been reported that the CHL content was decreased in plants subjected to herbicide stress [27], [28]. The current study also showed that the CHL a and b contents were significantly decreased in black nightshade treated with prometryn 3 d and 6 d after application. Application of prometryn in combination with GA_3_ caused a larger decrease in the CHL content compared with application of prometryn alone.

The soluble proteins in plants can enhance resistance to cellular stress conditions, and the amount of soluble protein directly affects metabolism [29]. Guy [30] concluded that an increase in the soluble protein content during stress appears to be an indicator of plant tolerance. We found that the soluble protein content was decreased in plants treated with prometryn applied alone or in combination with either GA_3_ or DPC. Compared with all other treatments, the application of prometryn in combination with GA_3_ resulted in the largest decrease in soluble protein content.

Under stress conditions, the formation of reactive oxygen species (ROS) is enhanced, thus inducing protective responses and cellular damage [31]. Membrane damage is caused by lipid peroxidation, which is in turn caused by increased concentrations of H_2_O_2_ [32], [33]. MDA is a product of lipid peroxidation in cell membranes [34], [35], and its content in vivo can indicate the extent of oxidative stress in plants and membrane homeostasis. We have demonstrated that herbicide treatments cause a dramatic increase in the leaf MDA content in black nightshade, indicating the presence of oxidation. Moreover, the exogenous application of prometryn plus GA_3_ was effective in increasing the levels of MDA in the leaves of black nightshade in a time-dependent manner. In contrast, we found that the MDA content in plants treated with prometryn plus DPC was lower than that in plants treated with prometryn alone 6 d after application. This result suggests that the prometryn treatment causes damage to the leaves and implies that GA_3_ could increase this damage when applied with prometryn.

The improvement of stress tolerance in plants is often related to antioxidant activities. An increased ROS level always triggers the upregulation of antioxidant enzyme activity, which in turn protects plants from oxidative stress [36], [37]. SOD, POD, and CAT are the most important detoxifying enzymes that promote ROS scavenging [38], [39]. SOD is responsible for the scavenging of toxic O^2−^ in different cellular organelles [40]. Furthermore, H_2_O_2_ might be detoxified by CAT, POD, and the ascorbate–glutathione cycle [41]. Researchers have found that the antioxidant enzyme activity in plants treated with herbicides decreases with time [42], [43]. Here, we also found that the activities of SOD, POD, and CAT in prometryn-treated leaves decreased over time. The antioxidant system in plants is a protective mechanism that confers tolerance to environmental stresses [44]. We demonstrated that the exogenous application of GA_3_ decreased the enzyme activities in black nightshade leaves, thus strengthening prometryn-induced oxidative damage. However, compared to plants treated with prometryn alone, higher enzyme activities were observed in plants treated with prometryn plus DPC.

In conclusion, GA_3_ effectively controls black nightshade. The combination of prometryn and GA_3_ positively reduced the herbicide tolerance of black nightshade at least in part by decreasing the content of leaf CHL and the soluble protein content, enhancing membrane lipid peroxidation and weakening the activities of SOD, POD, and CAT. The results presented in this study provide a basic understanding of the role of GA_3_ when applied in combination with prometryn and indicate its potential use in weed management.
